# Use of cellular phone contacts to increase return rates for immunization services in Kenya

**DOI:** 10.11604/pamj.2017.28.24.12631

**Published:** 2017-09-13

**Authors:** Evans Mokaya, Isaac Mugoya, Jane Raburu, Lora Shimp

**Affiliations:** 1 USAID’s Maternal and Child Survival Program (MCSP)/John Snow, Inc (JSI), Nairobi, Kenya; 2 Kisumu County Health Team/Ministry of Health, Kisumu, Kenya; 3 USAID’s Maternal and Child Survival Program (MCSP)/John Snow, Inc (JSI), Washington DC, USA

**Keywords:** Vaccination, immunization schedule, cell phones, defaulter tracking, Kenya

## Abstract

**Introduction:**

In Kenya, failure to complete immunization schedules by children who previously accessed immunization services is an obstacle to ensuring that children are fully immunized. Home visit approaches used to track defaulting children have not been successful in reducing the drop-out rate.

**Methods:**

This study tested the use of phone contacts as an approach for tracking immunization defaulters in twelve purposively-selected facilities in three districts of western Kenya. For nine months, children accessing immunization services in the facilities were tracked and caregivers were asked their reasons for defaulting.

**Results:**

In all of the facilities, caregiver phone ownership was above 80%. In 11 of the 12 facilities, defaulter rates between pentavalent1 and pentavalent3 vaccination doses reduced significantly to within the acceptable level of < 10%. Caregivers provided reliable contact information and health workers positively perceived phone-based defaulter communications. Tracking a defaulter required on average 2 minutes by voice and Ksh 6 ($ 0.07). Competing tasks and concerns about vaccinating sick children and side-effects were the most cited reasons for caregivers defaulting. Notably, a significant number of children categorised as defaulters had been vaccinated in a different facility (and were therefore “false defaulters”).

**Conclusion:**

Use of phone contacts for follow-up is a feasible and cost-effective method for tracking defaulters. This approach should complement traditional home visits, especially for caregivers without phones. Given communication-related reasons for defaulting, it is important that immunization programs scale-up community education activities. A system for health facilities to share details of defaulting children should be established to reduce “false defaulters”.

## Introduction

Vaccinating children is one of the most successful and cost-effective public health interventions for addressing childhood mortality and morbidity [1, 2]. In most countries, vaccination programs were launched in the 1980s to address the then six most common causes of childhood mortality: tuberculosis (TB), diphtheria, pertussis, tetanus, polio and measles. Despite significant progress in the 1980s in reducing the incidence of these six diseases, coverage levelled off or declined in some countries in the 1990s, including Kenya. Nonetheless, between 2000 and 2006, the Kenya immunization program reported a slow but steady improvement in coverage. This improvement was attributed to the roll out of the World Health Organization (WHO)'s Reaching Every District (RED) approach [3] in selected districts and improved funding from both the government and the GAVI Alliance. These gains were compromised, however, due to the 2007-08 political conflicts, the proliferation of districts with no clear capacity-building plans for new immunization managers and the abolition of cost share funds previously used to fund immunization operational costs. According to Kenya's Demographic and Health Survey [4], 77% of children nationally were fully immunized in 2008. Based on Kenya's Health Information System (HIS) reports from 2008 and 2013, fully immunized coverage had stagnated at 80% [5], with regional and district disparities in coverage reported. Whereas the national average was 80%, there were districts with FIC coverage as low as 30%. In addition to the low coverage, some districts had high drop-out rates between pentavalent1 and pentavalent3 doses, resulting in many children either partially or insufficiently immunized. Previous studies conducted in Kenya have reported varied reasons for this underperformance, including poor health-seeking behaviour or lack of knowledge of caregivers, inadequate demand generation activities, stock-outs of antigens at facilities, cold chain-related logistical problems (such as lack of gas, malfunctioning or absent equipment), inadequate access to hard-to-reach areas, poor defaulter tracing and/or inadequately trained staff coupled with irregular supportive supervision [6–8].

To address the defaulting of clients who already accessed vaccination services, this operations research in three selected districts in western Kenya tested the use of phone call reminders of return dates for caregivers who had brought their children for their first vaccines. This was to ensure that all children in contact with the facilities completed the immunization schedule. Given that household ownership and use of cellular phones in Kenya is more than 65% [9], there is potential for using phones to trace defaulters and to increase return rates for immunization. In addition, the study explored the common reasons that led caregivers to not complete the vaccination schedule (i.e. at birth, 6 weeks, 10 weeks, 14 weeks, 9 months and 18 months as outlined by the Unit of Vaccine and Immunization Services (UVIS)) [1]. In Kenya, data capture and management at the facility level are still paper-based. A name-based permanent register with details of the vaccinated children is maintained in all immunizing health facilities. Although detailed, this permanent register does not raise an alert whenever a child defaults and the process of generating a manual defaulter list is tedious and time-consuming. To address this challenge, this study introduced a diary (tickler system) with minimum details (name, village, vaccines due, telephone number, name of community health volunteer responsible for child, vaccination status) that could facilitate prompt identification and daily listing of defaulting, thereby aiding effective follow-up of the children by health workers. The study aimed to achieve the following objectives: test the feasibility of using cellular telephone contacts to trace immunization defaulters in three districts in Western Kenya; document lessons learned and challenges in using cellular telephone contacts to trace immunization defaulters; document barriers to continued utilization of immunization services in the three districts in Western Kenya; estimate the cost of implementing this strategy to inform future scale-up efforts at district level.

## Methods

This study involved a longitudinal pre and post-test design with both qualitative and quantitative arms. Twelve health facilities with high drop-out rates were purposively selected in four districts^2^ to participate in the study. Among the twelve facilities were two high-volume district hospitals, four sub-district hospitals, three health centres and three dispensaries. Both rural and urban populations were represented in the sample. The quantitative component of the study entailed: registration in the diary of children coming for their first vaccines, recording phone contact information of caregivers (or a relative or neighbour, if the caregiver did not have a phone), booking the child for the next visit and recording the name of the community health worker responsible for the child's residence. These details were also captured in the permanent register at the facility. For each visit, when the child was brought on the scheduled date, the health worker would tick the name of the child and transfer that name to the date in the diary that corresponded to the next appointment date. If the child did not come for the appointment, health workers would wait for two weeks before the caregiver was called and reminded of the missed appointment date. In addition, to be able to estimate the cost of tracking a defaulter, the health worker recorded the number of times each caregiver was called and the estimated duration of each call before the child was either brought back to continue with the schedule or his/her immunization status was established. The duration and the cost of the calls were also confirmed from the monthly post-paid bills sent to the program for payments. The qualitative component entailed asking caregivers who had missed the appointment date to give reasons (self-reported with necessary probing) for not honouring the return date and therefore defaulting.

**Analysis**: Data from the diaries was used to monitor continuation of vaccination services. A longitudinal follow-up of each child was done on a monthly basis. Children who started services in the facility, but did not complete the schedule, were considered to be defaulters. In this study, the difference between the number of children who received pentavalent1 and those who completed the schedule up to pentavalent3 is known as the drop-out. When this number is translated as a percentage of the number of the children who started the pentavalent series, the figure is referred to as the drop-out rate. The dropout rate for the period of the study was compared to the dropout rate for the same period for two consecutive years preceding the study. The number of children successfully tracked using phone calls was calculated, along with the number tracked by community health workers during the study period and aggregated by health facility. The cost to track a single defaulter by phone call was calculated and compared to the cost of tracking the same defaulter by community health workers. Reasons for defaulting were aggregated into five thematic groups: competing tasks; not knowing or forgetting the return date; fear of vaccines and side effects; cultural beliefs; vaccinated in other facilities.

## Results

**Figure 1:**
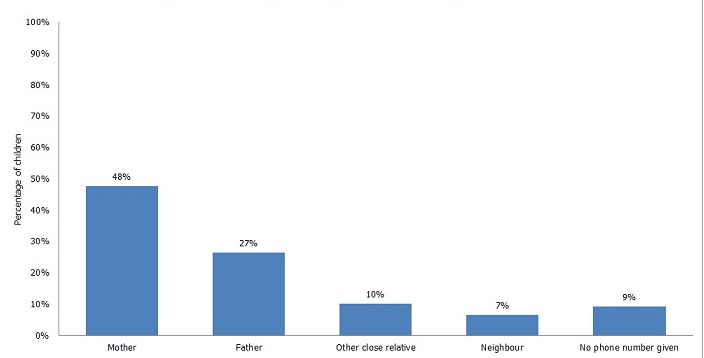
True vs apparent penta1 to penta3 defaulter rates, compared to basline rate for two years preceding the study

**Figure 2:**
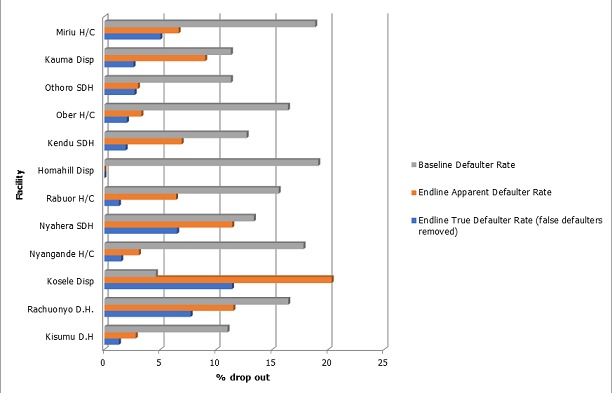
Relationship to child of cell phone owners whose contact information was given to health workers

A total of 5,908 children were enrolled and followed-up for a period of nine months in the 12 health facilities from May 2013 to March 2014, as shown in Table 1. The two urban district hospitals registered the largest number of children. Although Rabuor is a health centre, it enrolled the third highest number of children. The implementation rate of the study varied among the facilities. Nyahera SDH, Kauma Dispensary and Kosele Dispensary implemented the study for six months, due to interruptions of access to post-paid phone services, staff reluctance to implement the study in the initial phase and staff changeover. Ownership of a cell phone by a primary contact (father, mother or guardian) was well above 80% in all the facilities in the study. Although a rural/urban difference in ownership of phones was noted, the difference was minimal. It is significant that there were more urban women (47%) who owned phones compared to rural women (38%). Only 9% of caregivers did not provide any phone number (Figure 1). In order to facilitate easy tracking of children whose caregivers did not provide a reliable contact number, efforts were made to link them to responsible community health workers. The immunization defaulting rates (calculated using the formula described earlier) varied among the facilities. Compared to defaulter rates during the same period (May-March) in the two years preceding the study, all facilities except one reported a significant decline in the dropout rates, as shown in Figure 2. However, true defaulter rates (which account for children who were vaccinated elsewhere, such as outside the district) were lower in all facilities.

**Table 1:** Pentavalent 1 enrolment and use of defaulter tracking methods by facility

Facility Name	Pentavalent 1 Enrolment	Tracked by Phone	Tracked by Other Methods
Kisumu D.H.	1418	167	22
Rachuonyo D.H.	1194	156	30
Kosele Disp	90	19	2
Nyangande H/C	428	29	12
Nyahera SDH	529	79	19
Rabuor H/C	736	121	12
Homa-hills Disp	191	40	4
Kendu SDH	375	56	12
Ober H/C	297	36	6
Othoro SDH	297	37	8
Kauma Disp	151	24	4
Miriu H/C	202	21	10
**Total**	5908	785	141

A total of 785 defaulters were tracked using the provided phone contacts, whereas 141 were tracked by community health workers or other home visit approaches. Children tracked by the community health workers included those whose caregivers did not provide a phone number or gave incorrect numbers. On average, 1.5 calls lasting 2 minutes had to be made before the child's status was established and/or the child was brought back to the facilities for vaccination services. Therefore, the average cost of tracking a defaulter using the phone contacts among all the facilities in the study was Ksh 6 ($ 0.06). Assuming that the same child would default the subsequent three visits, the cost for tracking the child before he/she is fully vaccinated would average Ksh 18 per child ($ 0.18). On average, it took two minutes to track a defaulter. The cost of tracking a defaulter using community health workers was on average Ksh 125 ($ 1.25). In facilities that did not have committed community health workers, public health technicians and technologists were used to track defaulters. The cost of tracking a defaulter using a public health technician is on average Ksh 250 ($ 2.50) an aggregate cost that includes transportation and lunch. The time to travel from one house to another is not factored in. Other competing activities demanding the attention of the caregivers were the most commonly mentioned reasons for children missing the appointment dates in most of the facilities (Table 2). In three facilities (Ober, Kauma and Othoro), most of the defaulters were children who had been vaccinated elsewhere. A sick child or the fear of side effects (especially the pain and excessive crying following pentavalent vaccination) was the third commonly cited reason for missing a due date. Forgetting the appointment date was not a common reason. However, it is important to note that more defaulters from the two urban district hospitals cited this as a reason. Traditional or religious beliefs were not mentioned as a common reason for defaulting.

**Table 2:** Reason given by caregiver for defaulting on vaccination, by health facility.

		Ober H/COber H/C	MiriuH/C	OthoroSDH	KaumaDisp	KDH	RDH	Kosele	Nyangande	Nyahera	Rabuor	Homa-hills	Kendu SDH
**Competing tasks**	number	14	13	14	5	124	87	10	20	55	71	34	22
	%	33.3	41.9	31.1	31.3	65.6	46.8	47.6	48.8	56.1	53.4	77.3	32.4
**Not knowing or Forgot the return date**	number	2	2	1	1	17	21	1	1	1	4	2	6
	%	4.8	6.5	2.2	6.3	9.0	11.3	4.8	2.4	1.0	3.0	4.5	8.8
**Sick child and vaccine side effects**	number	6	6	10	4	18	35	3	9	18	20	8	15
	%	14.3	19.4	22.2	25.0	9.5	18.8	14.3	22.0	18.4	15.0	18.2	22.1
**Cultural / religious beliefs**	number	1	0	2	0	3	2	0	2	1	4	0	0
	%	2.4	0.0	4.4	0.0	1.6	1.1	0.0	4.9	1.0	3.0	0.0	0.0
**Vaccinated elsewhere**	number	19	10	18	6	17	38	7	6	21	30	0	19
	%	45.2	32.3	40.0	37.5	9.0	20.4	33.3	14.6	21.4	22.6	0.0	27.9
**No reason**	number	0	0	0	0	10	3	0	3	2	4	0	6
	%	0.0	0.0	0.0	0.0	5.3	1.6	0.0	7.3	2.0	3.0	0.0	8.8

## Discussion

This operations research set out to pilot the use of phone calls to increase the retention rates of children coming for immunization services. Utilization of services as defined by the drop-out rates (i.e. between penta1 and penta3) was a major challenge in the facilities identified to participate in the study. Prior to this pilot, several approaches had been used to track defaulters and bring them back to complete the immunization schedule. These approaches were predominantly based on home visits and included the use of community health workers, village elders and public health technicians who were expected to physically trace the whereabouts of the defaulting children, using information derived from the permanent registers. However, these approaches have not always been successful for three main reasons: low motivation among community health workers (lack of incentives), competing priorities for public health technicians and the lack of funds for lunches and transportation for public health technicians when tracking defaulters. Community health workers are not paid employees and hence are reluctant to perform defaulter tracking activities. Home visits are challenging and may involve the community health workers or public health technicians walking long distances (especially in rural areas) only to find that the client is not at home. Thus, the person would be required to visit the house more than once. This study was therefore developed to determine if a cheaper and more convenient alternative was possible, such as using mobile technology. Ownership of phones by caregivers/family was high in all the facilities and even more so in urban facilities-an important factor contributing to the feasibility of this approach. Concerns that rural caregivers would not have access to phones and therefore would not benefit from this approach were proven incorrect. However, the reluctance to share contact information, especially when the number did not belong to the caregiver, was an initial setback. To mitigate this, training was provided on how to communicate/ask for contact information and to reassure the caregivers that the number would not be shared with unauthorized persons or used for anything unwarranted.

As noted previously, the average cost of tracking a defaulter in all 12 facilities was estimated at Ksh 6 ($ 0.06). (If the child defaulted for all three subsequent visits, the cost would be Ksh 18 or $ 0.18.) In a busy health facility like Kisumu district hospital, an average of 30 children would default for more than two weeks, requiring tracing. This translated to at least Ksh 180 ($1.80) per month. For effective tracking of all defaulters in a year, the facility would therefore need to set aside more than Ksh 2160 ($ 21.60). In less busy facilities such as a dispensary, three children are estimated to default per month. The cost to track defaulters in such a facility would translate to Ksh 216/year ($ 2.16). On average, the time spent tracking defaulters in a busy district hospital and a dispensary would be 1 hour and 6 minutes per month, respectively. In the first two months of the study, some caregivers delayed coming for their appointment until they received the call from the health workers. In order to address this problem, health workers from the facility and the community health workers participating in the study were encouraged to counsel mothers on the benefits of honouring the appointment date and vaccinating children in a timely manner. Contrary to findings by a previous study [9], few caregivers in this study mentioned “forgetting the return date” as a reason for not honouring the return date (Table 2). However, the assumption that caregivers forget appointment dates has triggered the development and implementation of several SMS-based reminder platforms in various parts of the world [10–12]. Considering this study's findings, reminder SMS messages alone may not resolve the lack of continuity of participation in immunization services. Rather, this study supports calling the caregiver as an alternative or accompaniment to sending an SMS reminder. Through calling, the health worker is able to establish a personal relationship with the caregivers, understand the reasons behind missing the due date and negotiate with the caregivers on the most appropriate time and date to vaccinate the child. An SMS does not provide the health worker with the opportunity to get proper feedback from the client. Although calling is perceived to be expensive, this study provides evidence that the cost of implementing the call approach is feasible and that with proper planning and budgeting, facilities could save funds previously spent on tracking defaulters using the home visit approach.

The four most common reasons cited by caregivers for defaulting immunization were: competing tasks; the child was vaccinated in another facility within or outside the district; competing tasks (especially attending funerals and other social events) and afterwards fearing that the health workers will rebuke them for skipping the return dates; or the child was sick. Traditional beliefs did not feature strongly as a reason for defaulting immunization services. Although a sick child is not an absolute contraindication for vaccination, caregivers were apparently hesitant to bring their sick children for vaccinations. The pain and excessive crying following the pentavalent vaccination seem to also discourage some caregivers from bringing a child back to complete the schedule. Additional communications are needed to address this. A significant number of children thought to be defaulters were children who had been vaccinated in other facilities in the districts or in other neighbouring districts. These false defaulters, unless tracked and documented, contribute to incorrectly elevated defaulter rates, notably in urban facilities like Kisumu and Oyugis district hospitals. A major limitation of this study was that caregivers were not able to critique health worker practices, given that the calls/interviews were made by the same health workers or community health workers. Therefore, reasons such as long waiting times and poor attitudes of health workers cited in other literature were not noted in any of the facilities. Caregivers who provided phone contact information for their male spouses were more likely to respond to calls favourably and have their children vaccinated, which could reflect the decision-making power vested in male spouses in western Kenya. This finding needs further analysis, however, as children were not brought to the clinic by their fathers nor were females accompanied by their spouses. This implies low male involvement in the child's vaccination session. This finding is consistent with findings of low male involvement in matters related to antenatal care in Kenya [13].

In eight out of the 12 health facilities, community health workers were involved in asking for the phone contacts and in recording client details in the diary and permanent register. Compared to the other facilities where nurses fully implemented the study, the quality of the data and reporting were better in areas where community health workers were involved. This is partly because of the heavy burden on health workers for documentation of different health interventions offered in the maternal and child health (MCH) clinics. Community health workers, when well-oriented and supervised, could assist with documentation. This finding is consistent with the practice in Bondo district [14], where community health workers are assigned on a rotational basis to manage an exit desk in health facilities. At the exit desk, clients are triangulated and given return dates for antenatal care, preventing mother to child transmission of HIV, immunization and other services. As noted previously, the cost and time spent on tracking a defaulter by telephone is less than the cost of using a community health worker or public health technician to physically track defaulters. When budgeted, facilities can finance their operational costs for telephone reminders from the health sector support funds (HSSF). The telephone cost was so low that in the facilities where the post-paid lines were non-functional, health workers opted to use their personal lines or the facility phone. The implementation of this study faced several challenges, notably start-up delays and connectivity issues due to ceilings on the amount of funds that a facility could spend in a month for post-paid lines. The mobile phone provider used for the study did not ordinarily set limits on post-paid lines; however, the provider opted to set a ceiling that encompassed all 12 lines used in this study. This meant that if one facility spent the entire amount for the month, the lines for all 12 facilities were temporarily disconnected, thus affecting implementation across the facilities. Encouragingly, some health workers opted to use their personal lines to call when the post-paid lines were not available. When using their personal lines, health workers were encouraged to document details of the client and the duration of the call in a telephone log for inclusion in the analysis.

## Conclusion

The use of phone calls to track and communicate with defaulters contributed to the improved retention of clients for immunization in the intervention facilities. Considering phone ownership rates among caregivers and the low cost of calling clients, the use of phone contacts can be a cost-effective alternative to other defaulter tracking mechanisms practised in Kenya. To also improve participation in immunization, additional communication activities to encourage male involvement are potentially important for health programs to consider. As a result of this study, county managers in the intervention counties have been encouraged by the Kenya immunization program to incorporate this low-cost defaulter tracking mechanism as part of their service delivery and client interactions, along with the use of community resource persons. In addition, as approximately 40% of the defaulters in the intervention facilities were not real defaulters (i.e. the children were vaccinated elsewhere), it has been recommended that counties develop a system or platform-such as disaggregating children based on whether they are from the catchment area-for MCH staff to share information on children who transfer from other facilities. This information-sharing system between health facilities is critical to minimize potential wastage of facility resources on tracking children who are already vaccinated. Monthly facility in-charges meetings or/and the quarterly review meetings provide such a platform for sharing this information.

### What is known about this topic

- Between 2008 and 2013, the fully immunized child coverage rate in Kenya stagnated at around 80% nationally and as low as 30% in some districts. These low coverage rates were coupled with high drop-out rates between vaccines in a series;
- Previous studies link Kenya's high drop-out rates to poor health-seeking behaviour, lack of demand generation activities, stock-outs, cold chain-related logistical problems, malfunctioning equipment, inadequate access to hard-to-reach areas, poor defaulter tracing and inadequately trained staff coupled with irregular supportive supervision.

### What this study adds

- Tracking immunization defaulters through phone calls is a feasible and cost-effective alternative to the traditional home visit strategy, with phone calls costing on average $0.07 and two minutes per child for each missed visit;
- The most common reasons cited for missing an immunization appointment related to competing priorities and misconceptions about side effects or vaccinating sick children. Caregivers rarely stated that they had forgotten the appointment, contrary to previous studies. This finding suggests that phone calls in which healthcare workers can provide information about vaccination may be more effective than SMS reminders;
- A significant number of reported defaulters were “false defaulters” who had been vaccinated in another facility. This finding suggests a need for sharing information about defaulters between facilities.

## Competing interests

The authors declare no competing interest.
